# A Case of Concomitant Multiple Myeloma and Cirrhosis

**DOI:** 10.7759/cureus.44286

**Published:** 2023-08-28

**Authors:** Ziad Hindosh, Anil Potharaju

**Affiliations:** 1 Internal Medicine, University of Arizona College of Medicine - Tucson, Tucson, USA; 2 Internal Medicine, Banner Health, Tucson, USA

**Keywords:** hepatorenal damage, acute kidney injury, complex medical history, immunosuppression, cirrhosis, multiple myeloma

## Abstract

A 66-year-old Caucasian female with a recent history of COVID-19 (about one month prior to the current admission) and cirrhosis, presented with acute kidney injury (AKI) and worsening encephalopathy. Initial management focused on addressing her AKI, initially suspected to be secondary to volume depletion or hepatorenal syndrome (HRS) and confusion from hepatic encephalopathy. However, further evaluation unveiled a persistent serum protein gap, hypercalcemia, and significant non-albumin proteinuria, prompting a more comprehensive diagnostic approach. Subsequent investigations revealed a high serum free light chain ratio, positive serum protein electrophoresis, and monoclonal gammopathy, indicative of a plasma cell disorder. A bone survey did not indicate aggressive bone lesions, but a bone marrow biopsy confirmed multiple myeloma with approximately 10% kappa light chain-restricted plasma cells. Despite appropriate treatment, the patient's health continued to decline, and the patient was subsequently transitioned to comfort care.

While the relationship between cirrhosis and multiple myeloma remains to be fully understood, our case report explores four potential explanations: coincidental coexistence, cirrhosis as a risk factor for multiple myeloma, multiple myeloma as a risk factor for cirrhosis, or a shared predisposing condition.

## Introduction

Liver cirrhosis and multiple myeloma are both medical conditions that affect the elderly population. Although they usually manifest as distinct entities, rare instances arise where patients experience both conditions concurrently. This case report aims to emphasize the necessity for meticulous clinical assessment and a broad diagnostic approach when addressing patients with diverse symptoms, particularly within an aging demographic, and explore the reasons why the two conditions may coexist.

## Case presentation

A 66-year-old Caucasian female with a medical history of obesity (BMI: 34 kg/m²), cirrhosis, diabetes, hypertension, and asthma presented with acute encephalopathy. One month prior to the current hospitalization, she experienced acute hypoxic respiratory failure due to COVID-19. At that time, ascites and a platelet count of 92 k/uL were noted, and further inquiry revealed a cirrhosis diagnosis a few months earlier. The etiology was unclear. An ultrasound confirmed cirrhosis and portal hypertension. Initial liver function tests showed aspartate aminotransferase (AST) at 26 U/L, alanine transaminase (ALT) at 12 U/L, and mildly elevated alkaline phosphatase (ALP) at 229 U/L. Her calcium level was 10.6 mg/dL with an albumin level of 3.2 mg/dL and a total protein level of 7.9 g/dL; the protein gap was attributed to acute infection in the presence of liver disease. Upon resolution of acute respiratory failure, she was discharged with instructions for an outpatient cirrhosis evaluation. On discharge, her calcium level was 10 mg/dL, and urinalysis did not reveal proteinuria.

About a month later, she was readmitted with worsening encephalopathy, subjective fevers, and an abnormal urinalysis. Her mental state improved after receiving antibiotics and fluids in the emergency department. Upon admission, her creatinine increased from 1.1 mg/dL to 1.4 mg/dL, and her calcium levels remained between 9.5 and 10.6 mg/dL. Although the urinalysis indicated a urinary tract infection (UTI) and dehydration in the presence of hyaline casts, there was no proteinuria. Further testing for hypercalcemia was initiated. Alongside treatment for the UTI, she received fluids and albumin, which improved her creatinine to 1.2 mg/dL. However, hypercalcemia persisted, and both renal function and encephalopathy deteriorated after the initial improvement. Hepatorenal syndrome and hepatic encephalopathy were considered, leading to the initiation of lactulose and rifaximin.

**Figure 1:**
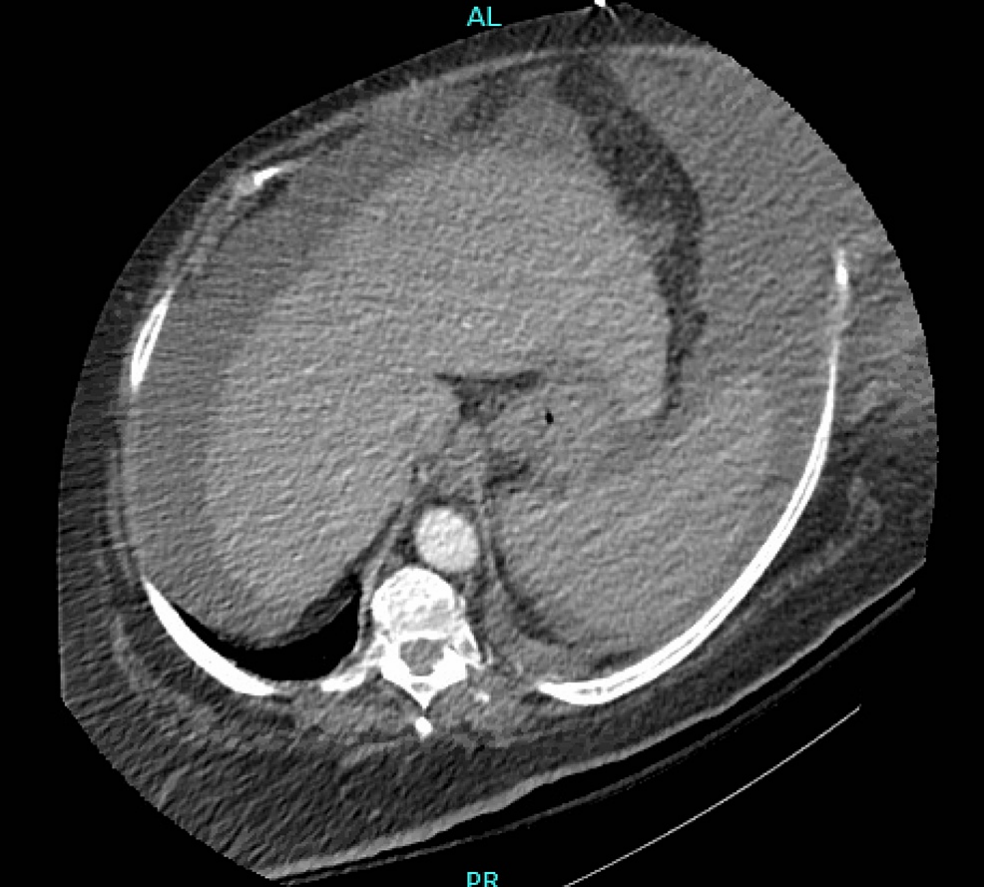
The patient's CT scan of the abdomen demonstrates cirrhotic liver

A CT of her abdomen revealed a nodular, cirrhotic liver with large-volume ascites, decreased lung volumes, and a small left-sided pleural effusion with compressive atelectasis (Figure 1).

The cirrhosis workup yielded inconclusive results, as her alpha-1-antitrypsin level, ceruloplasmin, anti-mitochondrial antibody, anti-liver-kidney microsomal (LKM) antibody, and antineutrophil cytoplasmic antibodies (ANCA) screen all returned negative. Iron studies ruled out hemochromatosis, with normal ferritin levels and low transferrin saturation. Smooth muscle antibody tested positive with a titer of 1:40, but AST and ALT were within the normal range, with mildly elevated ALP at 182 U/L. Her immunoglobulin G (IgG) was elevated to 2225, while her IgM was low at 24 mg/dL. Immunoglobulin A was normal, and complement factor 3 (C3) and C4 levels were low at 22 and 63 mg/dL, respectively. Hepatology was consulted, and the likely cause of cirrhosis was suspected to be nonalcoholic steatohepatitis (NASH), although the diagnosis could not be confirmed. However, given the absence of elevated AST and ALT levels and the patient's deteriorating condition, as well as the need for other urgent lab assessments (as discussed below), an immediate liver biopsy was not deemed necessary. Instead, it was recommended to schedule an elective esophagogastroduodenoscopy (EGD) and conduct further investigations for cirrhosis once the patient's condition stabilized.

The patient’s declining mental status persisted despite lactulose and rifaximin, and further tests such as an ascitic fluid analysis ruled out spontaneous bacterial peritonitis (SBP). Her rapidly deteriorating renal function, following her initial response, triggered further investigation. Her creatinine levels surged from 1.2 mg/dL to 4.5 mg/dL within a few days, despite her receiving octreotide, midodrine, albumin, and fluids. A repeat urine sodium level was not low, and a repeat urinalysis (UA) indicated moderate protein, both of which pointed to a diagnosis other than hepatorenal syndrome (HRS). Furthermore, neutrophil gelatinase-associated lipocalin (NGAL) levels were elevated to 3,448 ng/mL, suggesting the possibility of acute tubular necrosis (ATN). Considering the patient's ascites, bladder pressures were examined, revealing an intra-abdominal pressure of 21 mmHg, indicative of grade III intra-abdominal hypertension (abdominal compartment syndrome) as a potential cause of her acute kidney injury (AKI). Despite an urgent large-volume paracentesis, the patient's condition deteriorated, with her urine volume continuing to decline despite all the aforementioned interventions and diuretics. Consequently, dialysis was initiated.

**Figure 2:**
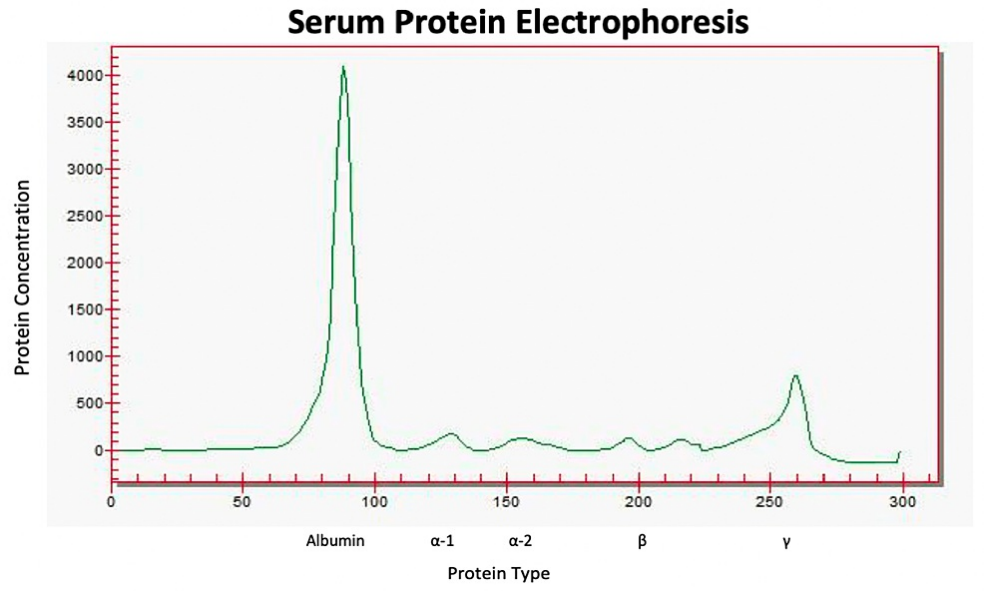
Serum protein electrophoresis demonstrating monoclonal gammopathy in the gamma region

In the meantime, a hypercalcemia workup revealed low parathyroid hormone (PTH) and normal vitamin D levels. The urine albumin to creatinine ratio was 1,188 mg/g, while the urine protein to creatinine ratio was 2,731 mg/g. With suppressed PTH, substantial proteinuria (albumin comprising 43% of total protein), persistent anemia, thrombocytopenia, and the serum protein gap, a plasma cell disorder diagnosis was pursued. serum protein electrophoresis (SPEP) indicated elevated Kappa light chain free at 777.53 mg/dL, Lambda light chain free at 143.91 mg/dL, and an elevated kappa/lambda free ratio at 5.4. A monoclonal spike (M spike) of 0.78 was noted, and serum immunofixation confirmed IgG kappa-type monoclonal gammopathy (Figure 2).

While imaging for lytic lesions was negative, her lactate dehydrogenase (LDH) and beta-2 microglobulin levels were elevated, and her bone marrow biopsy confirmed our suspicion as it showed a plasma cell neoplasm containing approximately 10% kappa light chain-restricted plasma cells, consistent with the diagnosis of multiple myeloma. Renal failure was attributed to possible cast nephropathy or amyloid protein accumulation in the context of ATN, though a renal biopsy was deferred due to thrombocytopenia and her worsening clinical condition. She was started on cyclophosphamide, bortezomib, and dexamethasone (CyBorD) but continued to decline and became hemodynamically unstable, at which point, following a conversation about goals of care, she was transitioned to comfort care. An autopsy was not performed.

## Discussion

Multiple myeloma is a neoplasm characterized by abnormal plasma cell proliferation in the bone marrow, primarily affecting older populations aged between 65 and 74 years [1]. The exact cause is unknown, but there are several associated risk factors, including sex, ethnicity, body mass index, and familial history [2-4].

The International Myeloma Working Group (IMWG) has established diagnostic criteria for multiple myeloma. Recent updates allow patients to receive treatment before symptomatic disease progression. The definition includes the presence of clonal bone marrow plasma cells exceeding 10% or biopsy-confirmed bony or extramedullary plasmacytoma, along with at least one CRAB (calcium elevation, renal insufficiency, anemia, and bone abnormalities) feature, which comprises hypercalcemia, renal insufficiency, anemia, or bony lesions [5]. Furthermore, new diagnostic criteria encompass a myeloma-defining event characterized by 60% or more clonal plasma cells in the bone marrow, a serum-free light chain ratio of 100 or higher, and the presence of more than one focal lesion on MRI measuring 5 mm or greater [5].

The unique coexistence of cirrhosis and multiple myeloma in this case prompts intrigue, considering the potential for one of four scenarios.

Firstly, this occurrence could simply be a coincidental improbable convergence of two independent diagnoses. The prevalence of multiple myeloma is approximately 9.3 per 100,000 women aged 65-69 years [6], while roughly 3% of women aged 65-74 years have been diagnosed with liver disease [7]. The simultaneous presence of these disorders is exceedingly rare. If this were the case, our report underscores the importance of avoiding fixation on a specific diagnosis (cirrhosis, in this instance), highlighting the value of diligently scrutinizing details that may diverge from the initial diagnosis.

Secondly, it raises the question of whether multiple myeloma predisposes individuals to cirrhosis. The extramedullary disease is an uncommon manifestation of multiple myeloma, occurring in less than 5% of cases at the time of diagnosis [8]. It typically involves the skin, soft tissues, paraspinal area, lymph nodes, and liver. While liver infiltration has been documented, multiple myeloma is not a known cause of cirrhosis. Additionally, liver infiltration tends to manifest as the disease advances or during relapse, not at the initial diagnosis stage and usually presents with hepatomegaly, jaundice, ascites, or biliary obstruction [9].

Thirdly, the case suggests the possibility that cirrhosis may predispose patients to other malignancies. While it is well established that cirrhosis predisposes individuals to hepatocellular carcinoma, the relationship between chronic liver disease and multiple myeloma remains ambiguous. Nevertheless, there are similarities in immunoregulation between these conditions, suggesting a potential link. Prior studies have explored the association between chronic liver disease and multiple myeloma, but the exact cause remains unclear. There are similarities in immunoregulation between autoimmune diseases and chronic active hepatitis, as patients with chronic liver disease often exhibit elevated levels of serum IgG, IgA, and IgM [10]. Various factors contribute to the increased production of immunoglobulins in damaged livers, such as impaired reticuloendothelial function and the inability to eliminate antigens and endotoxins in the portal system [10-11]. Evidence suggests that short-lived suppressor cell activity also plays a role in enhancing IgG synthesis. Hyperimmunoglobulinemia is not exclusive to autoimmune cases and can be seen in a variety of liver disorders, including chronic active hepatitis, alcoholic cirrhosis, and obstructive jaundice resulting from biliary strictures or stones [12].

Lastly, the coexistence of both cirrhosis and multiple myeloma in the same patient prompts consideration of a shared etiological factor. This common cause may arise from shared genetic, environmental, or lifestyle factors that predispose an individual to both diseases. While age and obesity both predispose to both of these conditions, given the rarity of simultaneous cirrhosis and multiple myeloma, exploring other potential causes such as chronic inflammation, immune dysregulation, genetics, or exposure to specific toxins needs to be considered to reveal previously unidentified risk factors as these may have preventative, diagnostic, or therapeutic implications.

## Conclusions

The coexistence of multiple myeloma and liver cirrhosis in our patient calls for further exploration of potential pathophysiological links and the development of effective diagnostic and therapeutic strategies. The dampened immune system resulting from cirrhosis might allow the proliferation of cancerous cells, although the exact mechanisms remain elusive. Alternatively, multiple myeloma may be an as-yet-unknown cause of cirrhosis. In the authors’ opinion, based on existing knowledge, the individual had two conditions with some shared risk factors, such as age and obesity. This underscores the importance of maintaining a high index of suspicion and considering the possibility of multiple diseases when clinical presentations are atypical. While Occam's Razor suggests seeking the simplest explanation that accounts for all symptoms, our case demonstrates the importance of considering multiple simultaneous pathologies, as per Hickam's Dictum, especially when the clinical course doesn't align.
